# Multi-Degree-of-Freedom Stretchable Metasurface Terahertz Sensor for Trace Cinnamoylglycine Detection

**DOI:** 10.3390/bios14120602

**Published:** 2024-12-09

**Authors:** Huanyu Li, Wenyao Yu, Mengya Pan, Shuo Liu, Wanxin Nie, Yifei Zhang, Yanpeng Shi

**Affiliations:** School of Integrated Circuits, Shandong University, Jinan 250100, China

**Keywords:** terahertz, stretchable metasurface, molecular fingerprint sensor, magnetic dipole resonance, all-dielectric metasurface, cinnamoylglycine

## Abstract

Terahertz (THz) spectroscopy, an advanced label-free sensing method, offers significant potential for biomolecular detection and quantitative analysis in biological samples. Although broadband fingerprint enhancement compensates for limitations in detection capability and sensitivity, the complex optical path design in operation restricts its broader adoption. This paper proposes a multi-degree-of-freedom stretchable metasurface that supports magnetic dipole resonance to enhance the broadband THz fingerprint detection of trace analytes. The metasurface substrate and unit cell structures are constructed using polydimethylsiloxane. By adjusting the sensor’s geometric dimensions or varying the incident angle within a narrow range, the practical optical path is significantly simplified. Simultaneously, the resonance frequency of the transmission curve is tuned, achieving high sensitivity for effectively detecting cinnamoylglycine. The results demonstrate that the metasurface achieves a high-quality factor of 770.6 and an excellent figure of merit of 777.2, significantly enhancing the THz sensing capability. Consequently, the detection sensitivity for cinnamoylglycine can reach 24.6 µg·cm^−2^. This study offers critical foundations for applying THz technology to biomedical fields, particularly detecting urinary biomarkers for diseases like gestational diabetes.

## 1. Introduction

The detection of cinnamoylglycine, a metabolite of cinnamic acid, holds significant promise for biomedical applications. Cinnamoylglycine is primarily produced through the metabolism of dietary cinnamic acid by gut microbiota and excreted in urine [1,2,3]. It has been suggested as a potential marker of gut health, reflecting the inhibitory effects of gut microbiota on pathogens [4]. Moreover, urinary cinnamoylglycine levels have been closely associated with various metabolic disorders, particularly showing a significant decrease in gestational diabetes mellitus (GDM) patients [5,6,7,8,9]. GDM is a common pregnancy complication characterized by increased maternal insulin resistance. It can lead to severe complications such as preeclampsia, preterm delivery, and fetal macrosomia [8,9], and it markedly increases the risk of developing type 2 diabetes later in life [9]. As changes in urinary cinnamoylglycine concentrations can reflect early signs of metabolic disturbances, it has been considered a practicable biomarker for diseases. Nevertheless, current identification methods, such as liquid chromatography–tandem mass spectrometry, are highly sensitive but limited in their widespread clinical application due to complex sample pretreatment processes and high costs [4,10].

To address the limitations of current detection methods, terahertz (THz) spectroscopy has emerged as a promising analytical tool. Spanning frequencies between 0.1 and 10 THz [11], it is a non-ionizing, non-destructive technique capable of penetrating non-conductive materials without damaging samples [12]. Due to these unique properties, THz spectroscopy excels in molecular fingerprinting of chemical and biological substances [11,12,13,14,15]. Its sensitivity to the absorption features of hydrogen bonds, molecular vibrations, and rotational modes in the THz band [16] further expands its potential for highly selective molecular detection. This capability has wide-ranging applications in biomedicine, imaging, and food safety [17,18]. Despite its advantages, the sensitivity of THz spectroscopy in trace analysis remains a significant challenge. The long wavelength of THz waves (30 µm to 3000 µm), while target analytes are typically far below the micrometer scale, weakens interactions and limits sensing performance [19]. This limitation hampers the broader application of THz technology in trace detection [20].

Therefore, enhancing its sensitivity, particularly for trace analysis, has become a key area of research. Several approaches have been proposed to improve detection performance, including surface plasmon resonance, nanoantenna metamaterials, metal slot arrays, and electromagnetic subwavelength structures, which strengthen the interaction between THz waves and analytes [21,22,23,24,25,26,27,28,29,30,31,32,33,34,35]. Nonetheless, metal-based techniques suffer from significant energy losses, particularly in detecting low-concentration analytes, limiting improvements in sensitivity [26]. Consequently, researchers have shifted their focus to all-dielectric metamaterials, utilizing the Mie resonance to more effectively manipulate interactions between electromagnetic waves and materials. This approach minimizes energy dissipation and increases the sensor’s quality factor (Q-factor), thereby significantly enhancing detection sensitivity [27,28,29]. Nevertheless, this method still faces challenges in accurately characterizing the broad absorption spectrum of trace analytes. Recently, an approach has been proposed that employs multiple metasurfaces with unit cell structures of varying sizes or changes in the incidence angle of electromagnetic waves. This strategy generates a series of resonance peaks, enhancing broadband interactions between electromagnetic waves and matter [32,33,34,36,37,38]. These interactions ultimately reflect trends in the extinction coefficient of the analyte. Despite various schemes employed in the metasurfaces, including the use of varying incident angles and unit cell geometrical dimensions, each method has its own limitations. The use of different incident angles complicates the design of optical paths during experiments, whereas incorporating varied unit structures demands highly precise fabrication techniques.

To further address the limitations of traditional THz spectroscopy while reducing the requirements for process precision, we propose a multi-degree of freedom. The proposed design is a periodically symmetric polydimethylsiloxane (PDMS) quadruple structure that supports magnetic dipole (MD) resonance. This sensor, which is relatively easy to manufacture and measure [39], consists of a PDMS substrate with clusters of four periodically arranged PDMS cubes, achieving a high Q-factor of 770.6 and a figure of merit (FoM) of 777.2. By leveraging its stretchable dielectric structure, the sensor enables dual modulation through stretching along the x-direction (100% to 130%) or adjusting the THz wave incidence angle (0° to 15°). These methods simplify the detection setup by overcoming the optical path complexity associated with traditional angle multiplexing while also providing efficient and dynamic tuning of the resonance frequency across a wide range. This capability allows the resonance envelope to overlap with the absorption resonance of cinnamoylglycine, enabling a detection limit of 24.6 µg·cm^−2^ for cinnamoylglycine. By introducing a multi-degree-of-freedom tuning approach, this study offers a convenient and versatile platform for high-sensitivity detection of trace THz molecular fingerprints while also providing new strategies for THz sensing.

## 2. Materials and Methods

**Figure 1:**
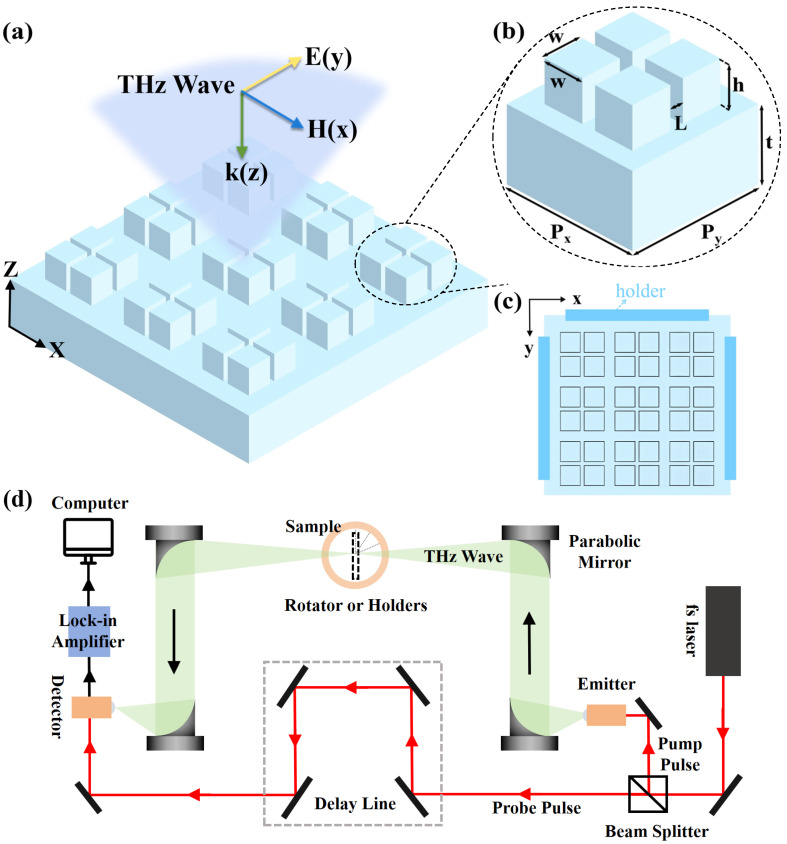
(**a**) Structural view of an all-dielectric metasurface, showing the periodic arrangement of pure PDMS cubic clusters. (**b**) Unit cell of the periodic structure. (**c**) Schematic of the structure of the holder with the y-axis fixed and moving along the x-axis. (**d**) THz-TDS system based on a photoconductive antenna.

The proposed all-dielectric metasurface, illustrated in Figure 1a, consists of cube clusters made from PDMS, arranged periodically in a square lattice on a PDMS substrate. In the THz regime, PDMS exhibits a relative permittivity of 2.35 and a loss tangent of 0.04 [40]. It also maintains excellent stability under normal conditions, with both thermal and mechanical aging having minimal effects within 10,000 h of use [41,42]. The geometrical parameters of the metasurface, depicted in Figure 1b, include lattice constants Px = Py = 410 µm, substrate thickness t = 300 µm, cube side length w = 150 µm, height h = 170 µm, and inter-cube spacing L = 30 µm. The y-polarized THz wave is incident vertically along the z-direction. By tuning the incident angle of the light source or applying strain to the substrate, the resonance frequency in the transmission spectrum can be shifted. As a result, an envelope curve is formed that covers the absorption band of the analyte. To apply strain to the substrate and achieve the frequency shift, a support structure consisting of four holders is positioned at the bottom layer of the tetrameric configuration, as shown in Figure 1c. In the experiment, the device is characterized using a photoconductive antenna-based THz-TDS system, with the setup shown in Figure 1d. The incident angle is varied by rotating the sample, while strain is applied by adjusting the holder positions to stretch the substrate.

To evaluate the performance of the proposed sensor, the optical properties of the metasurface were simulated using the 3D finite-difference time-domain method. In this simulation, a single unit cell was analyzed, with both the incident angle and the degree of stretching varied independently. Periodic boundary conditions were imposed along the x- and y-axes, while a perfectly matched layer boundary condition was employed along the z-axis. The stretching factor S, defined as the ratio of the stretched dimension to the original dimension, quantifies the degree of deformation. The top and bottom layers were cured under different conditions, resulting in a significant contrast in their elastic moduli [43]. This disparity in mechanical properties leads to distinct performance characteristics between the two layers, particularly under mechanical strain. During stretching along the x-direction, deformation is primarily confined to the substrate, with minimal effect on the top layer, which can be neglected. This ensures the preservation of structural integrity and stable performance under mechanical strain. As the stretching factor S increased, the unit cell dimension P_x_ and the inter-cube spacing L varied proportionally along the x-axis, while parameters along the y-axis remained largely unaffected, and the dimensions of the upper cube clusters were kept fixed.

## 3. Results and Discussion

**Figure 2:**
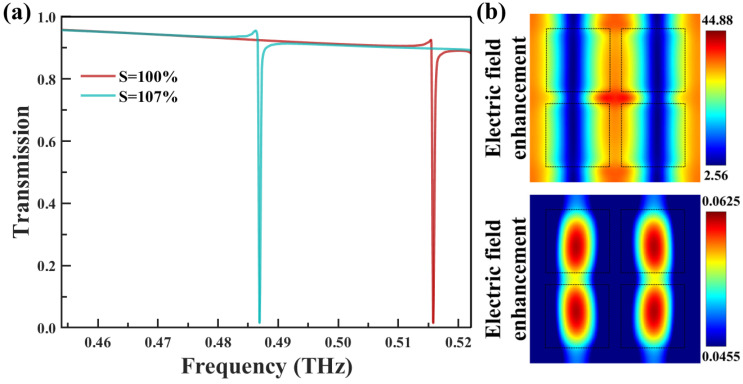
(**a**) Metasurface transmission spectra at stretch factor S = 100% and S = 107%. (**b**) Electric and magnetic field distributions measured at the surface of the PDMS substrate at stretch factor S = 100%.

To gain deeper insights into the physical properties, the THz response and transmission behavior of the metasurface were systematically analyzed under varying deformation levels. The physical mechanism behind the sharp resonance drop, which can be explained by the Fano resonance principle [44], is illustrated in Figure 2. Under perpendicular incidence of the THz wave, a pronounced resonance occurred at 0.516 THz when S = 100%, as depicted in Figure 2a. As stretching factor S increased to 107%, the resonance frequency shifted towards lower values due to the increased periodicity, eventually reaching 0.487 THz. At this point, the resonance frequency of the y-polarized wave matched the absorption peak of cinnamoylglycine while exhibiting a Q-factor of 770.6, defined as Q = f_0_/Δf, where f_0_ is 0.487 THz and Δf is 0.632 GHz. The electric and magnetic field distributions in the x–y plane are illustrated in Figure 2b. This resonance phenomenon originates from the excitation of the MD mode, manifesting as a collective response of four longitudinal MDs [45]. The electric field is primarily concentrated in the central region of the clusters, indicating the excitation of MD resonance in this region, which enhances the interaction between the incident THz wave and the analyte. Similarly, the magnetic field distribution reveals the presence of MD resonance. By leveraging these distinct field distributions, the sensor can more effectively detect the analyte, leading to enhanced sensitivity and specificity in the detection process.

**Figure 3:**
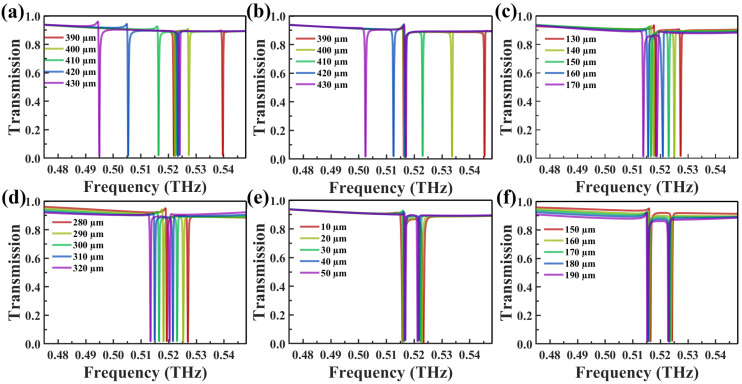
(**a**) Effect of varying periodicity in the x-direction P_x_ on the transmission curve. (**b**) Effect of varying periodicity in the y-direction P_y_ on the transmission curve. (**c**) Impact of varying cube size w on the transmission curve. (**d**) Influence of varying substrate thickness t on the transmission curve. (**e**) Effect of varying inter-cube cluster distance L on the transmission curve. (**f**) Effect of varying cube cluster height h on the transmission curve.

To achieve higher quality factors for the metasurface peaks and ensure better adaptation to biosensing applications, this study optimized several geometrical parameters of the unit cell, including the lattice constants (P_x_, P_y_), substrate thickness (t), cube side length (w), cube height (h), and inter-cube spacing (L). These parameters were varied in steps of 10 µm, and the impact on the transmission peaks is shown in Figure 3. The simulation results show that variations in the lattice constants (P_x_, P_y_) primarily drive the resonance frequency shift, whereas changes in the cube dimensions (t, w) induce comparatively smaller shifts. In contrast, the spacing (L) and height (h) exert minimal influence on the frequency shift but have a pronounced effect on the resonance sharpness and quality factor. After considering experimental constraints and the required performance for biosensing, the final optimized values were selected as P_x_ = P_y_ = 410 µm, t = 300 µm, w = 150 µm, h = 170 µm, and L = 30 µm. This optimization ensures that the metasurface achieves a balance between sensitivity, resolution, and structural stability, thereby maintaining optimal performance in biosensing applications, as required for practical deployment.

**Figure 4:**
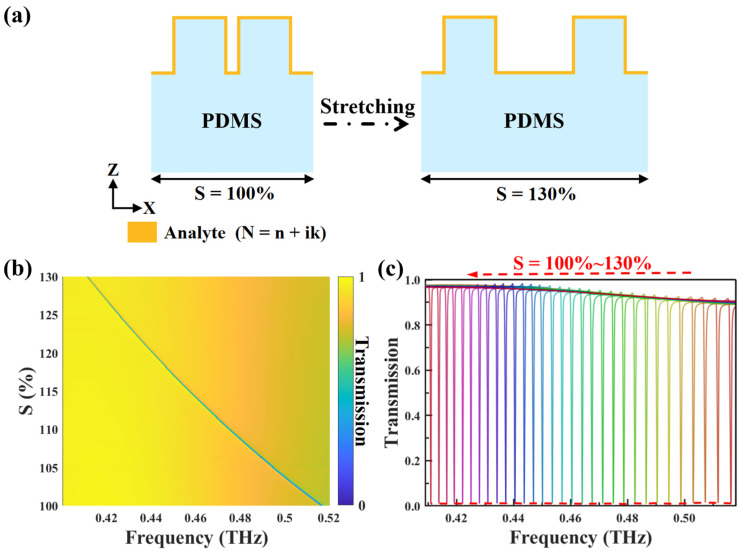
(**a**) Schematic diagram of the metasurface structure with the attached analyte as the stretching factor S varying from 100% to 130%. (**b**) Two-dimensional contour plot of the transmittance as a function of the stretching factor and frequency. (**c**) Normalized transmission spectra without analyte (stretching factor ranging from 100% to 130%).

Deeper insights into its properties were obtained by investigating the THz response under different degrees of stretching. The geometric deformation of the THz metasurface unit cell during the stretching process is schematically illustrated in Figure 4a. By stretching the unit cell along the x-axis, the structure undergoes controllable deformation while maintaining its dimensions in the other directions. As the stretching factor S increases from 100% to 130%, the distance from the center of the cube to the center of the structural unit expands from 90 μm to 117 μm. Meanwhile, the corresponding unit cell length P_x_ increases from 410 μm to 533 μm. This structural deformation caused by stretching modulates the incident THz wave, leading to distinct transmission behaviors that vary with the stretching factor S. As illustrated in Figure 4b, the transmittance is plotted as a function of the metasurface stretching factor S, indicating multiple narrow transmission bands. The frequency of the transmission peaks decreases linearly and monotonically as the stretching factor increases from 100% to 130%. To more clearly illustrate the influence of stretching modulation, the transmission spectra curves under different stretching factors are presented in Figure 4c. It can be observed that the incident THz wave is modulated to form a series of sharp resonances in the transmission spectra. The resonance frequency tuning range induced by stretching covers 0.516 THz to 0.411 THz, which aligns with the absorption frequency of the target analyte, cinnamoylglycine. Establishing this frequency matching is crucial for enhancing the selectivity of the sensing detection, as it ensures that the metasurface device can capture the fingerprint absorption of the analyte. It is worth noting that while the resonance peak position shifts, the peak intensity remains essentially unchanged. This stability and consistency of the spectral response are essential for ensuring the repeatability and reliability of the sensor.

**Figure 5:**
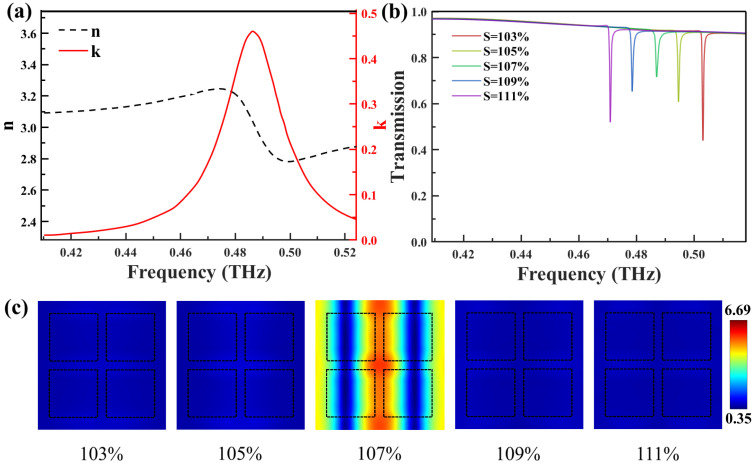
(**a**) Refractive index and complex refractive index of cinnamoylglycine in the THz band. (**b**) Stretch factor dependent transmission spectra of 0.6 µm thick cinnamoylglycine on a metasurface. (**c**) The electric field distribution measured at the substrate surface in the x–y plane at 0.487 THz for specific stretching factors S, corresponding to the transmission spectra shown in (**b**), respectively.

To comprehensively evaluate the performance of the designed flexible THz sensor, a thin film of cinnamoylglycine was applied to the device surface. The complex refractive index of cinnamoylglycine is shown in Figure 5a, with the data extracted using the Fresnel formula [46]. It can be observed that the extinction coefficient k reaches a maximum at 0.487 THz, marking the fingerprint absorption peak of cinnamoylglycine molecules. The real part, n, indicates the refractive index, while the imaginary part, k, represents the extinction coefficient. The transmission spectrum curves measured under different stretching factors after coating a 0.6 μm thick layer of cinnamoylglycine are presented in Figure 5b. As the stretching factor gradually increases, the transmission peak position undergoes a noticeable redshift, while the peak amplitude also exhibits an upward trend from 43.45% to 60.48%, eventually reaching 71.45%. It is particularly noteworthy that when the stretching factor is 107%, the resonance peak reaches its maximum at 0.487 THz. This corresponds to the maximum in the extinction coefficient curve of cinnamoylglycine. To gain a deeper understanding of the sensing enhancement mechanism, the near-field distribution characteristics of the device were further analyzed under different stretching states at 0.487 THz. The electric field distribution maps of the metasurface structure, as the stretching factor varies from 103% to 111%, exhibit clear changes, as shown in Figure 5c. It can be observed that at a stretching factor of 107%, the electric field intensity on the device surface is maximally enhanced. At this point, the strong electric field signals induced between adjacent cube clusters create a unique pattern of localized electric field enhancement, boosting the interaction strength between molecules and THz waves. In contrast, under other stretching states, the localized electric field enhancement effect is significantly weakened. The resonance frequencies at these stretching factors are far from the peak frequency of the extinction coefficient, which accounts for this weakening. Such an electric field distribution can enhance the wave–matter interactions around the resonance frequency, thereby boosting the sensing capability of the metasurface.

**Figure 6:**
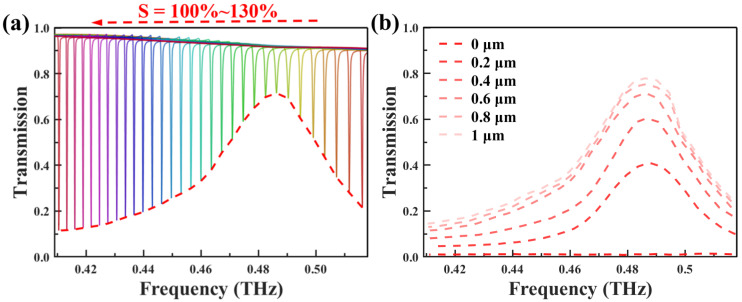
(**a**) Normalized transmission spectra of 0.6 µm thick cinnamoylglycine with stretch factors ranging from 100% to 130%, the S values increasing by 1% between each curve. The corresponding envelopes have been plotted with red lines. (**b**) Transmission envelope curves for different thicknesses of analytes.

As demonstrated in Figure 6, the THz metasurface sensor clearly distinguishes cinnamoylglycine from other substances through a geometric parameter multiplexing strategy, leveraging the distinctive absorption characteristics of different materials in the THz range. A series of normalized transmission spectrum curves, obtained through continuous stretching modulation, is shown in Figure 6a. Specifically, the stretching factor was gradually increased in 1% increments, with transmission changes recorded within the stretching range of 100% to 130%. By tracking the transmission peak positions, a distinct envelope curve, indicated by the red dashed line, was observed. Notably, the envelope curve reaches its maximum near 0.487 THz. It closely aligns with the cinnamoylglycine extinction coefficient curve in Figure 5a, indicating that the PDMS-based metasurface fingerprint sensor can accurately identify cinnamoylglycine. Simultaneously, to quantitatively evaluate the sensor capability for the absorption characteristics of trace analytes, the influence of analyte film thickness on the device transmission characteristics was investigated. As shown in Figure 6b, as the thickness of the cinnamoylglycine thin film gradually increases from 0.2 μm to 1 μm, the transmission peak amplitude of the device at 0.487 THz monotonically rises from 41.06% to 77.86%. The detection limit, determined by σ = ρ × h with an analyte volume density of ρ = 1.23 g/cm^3^ and a minimum layer thickness h = 0.2 μm, is 24.6 μg/cm^2^. For comparison, the transmission envelope curve without the analyte coating is also presented, maintaining an amplitude close to zero throughout. This result fully demonstrates that the designed sensor exhibits a clear response to changes in analyte thickness, paving the way for quantitative analysis. To comprehensively evaluate the sensor performance, FoM was introduced as a key performance indicator. It is defined as FoM = S/FWHM = S × Q/f_0_ [47]. Therefore, a sensor with an FoM of 777.2 was obtained, demonstrating superior performance. This value indicates that the device achieves excellent levels in multiple performance indicators such as sensitivity, selectivity, and Q-factor, making it a high-performance THz sensor with broad application prospects.

**Figure 7:**
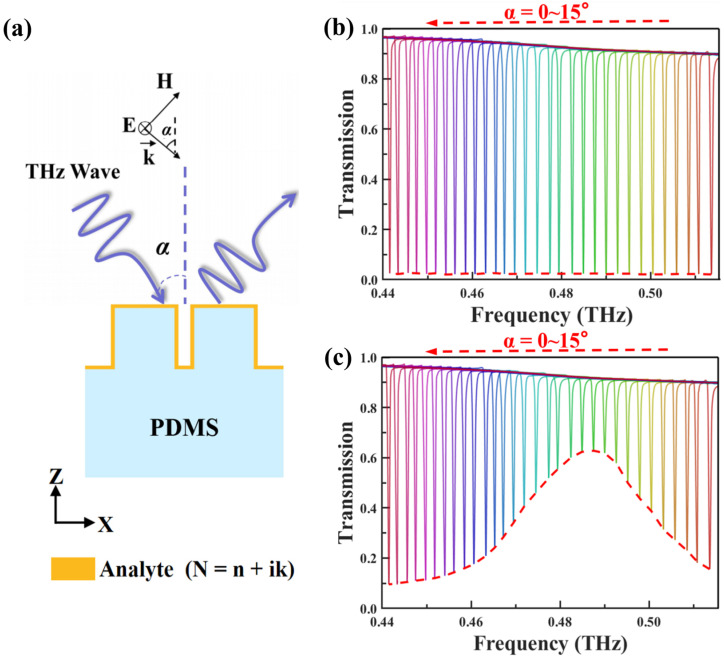
(**a**) Schematic diagram of angle multiplexing in the x–z plane of the metasurface. (**b**) Normalized transmission spectra without any analyte, with angles of incidence ranging from 0° to 15°, the values increasing by 0.5° between each curve. (**c**) Integrated transmission spectrum of a 0.6 µm thick cinnamoylglycine film with incidence angles ranging from 0° to 15°, the values increasing by 0.5° between each curve. The corresponding envelope has been plotted with a red line.

Further discussion was made regarding the influence of incident angle variations on the detection performance of the flexible THz sensor, with a focus on the modulation effect of incident angle α on the metasurface, which varied from 0° to 15°. The structural parameters of the tetramer metasurface were kept unchanged, and the corresponding transmission spectra were characterized at a stretching factor S = 100%, as shown in Figure 7a. The transmission spectrum curves obtained within the incident angle range of 0° to 15° are presented in Figure 7b. The results indicate that each incident angle corresponds to a unique narrow-band transmission peak. As the incident angle increases, the center frequency exhibits a significant monotonic decreasing trend. Meanwhile, the device maintains a constant minimum transmission value and spectral line width in the absence of the analyte. This observed frequency shift can be attributed to the disruption of structural symmetry conditions caused by changes in the incident angle. As the perturbation increases, the frequency shift broadens [48]. Additionally, modifying the parameters of the four cubes can lead to shifts in the MD resonance frequency [49]. When these different effects combine, they contribute to the formation of clusters in the transmission spectrum, covering a wide range of frequencies that include the absorption frequencies of the analyte. This combination of results allows for targeted identification of the samples. To intuitively evaluate the detection performance, a 0.6 μm thick cinnamoylglycine thin film was deposited on the metasurface, and the results are shown in Figure 7c. Due to the influence of the optical loss of cinnamoylglycine, the transmission envelope curve reaches a maximum value at 0.487 THz, with a corresponding transmission as high as 62.8%. This peak value precisely corresponds to the characteristic absorption frequency of cinnamoylglycine molecules. This phenomenon further validates the application potential of the developed flexible THz metasurface in molecular fingerprint sensing. Notably, unlike other THz sensing schemes that often require complex modulation over a large angular range [34,35,50], the metasurface proposed only requires only a limited scan within 0° to 15°. This design approach significantly simplifies the optical path design process during measurement and improves detection efficiency, offering a more intuitive and convenient solution for practical applications.

To compare the performance of the proposed metasurface with previous designs, key parameters are summarized in Table 1. The proposed structure combines angular and geometric multiplexing approaches, which helps to reduce or even avoid the complexity of optical path setup in practical applications. This multi-degree-of-freedom design not only simplifies the optical system but also enhances the flexibility of the device, making it more adaptable to varying experimental conditions.

To compare the performance of the proposed metasurface with previous designs, key parameters are summarized in Table 1. As shown in Table 1, the proposed structure outperforms previous designs in terms of both Q-factor and FoM, demonstrating significant improvements in performance. The proposed structure combines angular and geometric multiplexing approaches, which helps to reduce or even avoid the complexity of optical path setup in practical applications. This multi-degree-of-freedom design not only simplifies the optical system but also enhances the flexibility of the device, making it more adaptable to varying experimental conditions. Furthermore, the multiplexing range in our work is narrower than those in previous studies, allowing for high detection sensitivity while simultaneously reducing the practical detection time. In the angular multiplexing scheme, each spectrum takes 2–3 min, with the total time under 45 min. The stretching multiplexing scheme, despite the larger range, is faster and requires fewer adjustments, resulting in comparable or shorter total measurement time. This compact scanning range ensures efficient data collection without compromising the resolution and quality of the terahertz spectra.

## 4. Conclusions

In conclusion, this study presents a multi-degree-of-freedom THz sensor for the trace detection of cinnamoylglycine, a potential urinary biomarker for GDM, based on an all-dielectric metasurface. The proposed sensor, with a fully PDMS structure of periodic cube clusters on a substrate, achieves a high Q-factor of 770.6 and an FoM of 777.2. By utilizing the properties of PDMS, the sensor combines stretchable dielectric structures and angle scanning, enabling stretch modulation and limited angular multiplexing. This design allows tuning spectral transmission peaks and recovering broadband fingerprint signals from trace analytes. As a result, it achieves high-sensitivity detection, especially for ultrathin trace analytes in various physical states or forms. These methods greatly reduce the complexity of optical path design during the detection process, simplifying practical operations while ensuring sensitivity compared to traditional methods. Given the health risks associated with GDM, the proposed flexible THz metasurface sensor offers a rapid, convenient, and accurate alternative to current diagnostic methods. To further advance its practical utility, future work will focus on optimizing the structural design to improve adaptability, enhancing sensitivity to a wider range of molecular analytes, and addressing scalability for broader practical applications. Furthermore, the multi-degree-of-freedom modulation strategy enhances the versatility of THz sensors, broadening their biomedical applications and laying a foundation for future research in THz spectroscopy and diagnostics.

## Figures and Tables

**Table 1:** Comparison of multiplexing modes and ranges of existing metasurfaces and the proposed structure.

Ref.	Structure	Working Band	Q	FoM	Multiplexing Mode	Range of Multiplexing (Δ)
[37]	Pair cuboids	THz	140	11.1	Incident angle	0°~40°, 0°~30°
[38]	Pair pillars	Mid-infrared	110	-	Incident angle	1°~70°
[50]	Triangular tetramers	THz	231	609	Incident angle	13°~62°
[51]	Nanodisks array	Mid-infrared	>160	>33	Fermi effect	0.30 eV~0.72 eV
This work	Stretchable PDMS metasurface	THz	770.6	777.2	Incident angle and geometry	100%~130%, 0°~15°

## Data Availability

The data underlying the results presented in this paper may be obtained from the authors upon reasonable request.
